# Implementation and evaluation of a nurse-led decision-coaching program for healthy breast cancer susceptibility gene (BRCA1/2) mutation carriers: a study protocol for the randomized controlled EDCP-BRCA study

**DOI:** 10.1186/s13063-020-04431-x

**Published:** 2020-06-08

**Authors:** A. Isselhard, M. Töpper, B. Berger-Höger, A. Steckelberg, H. Fischer, F. Vitinius, K. Beifus, J. Köberlein-Neu, R. Wiedemann, K. Rhiem, R. Schmutzler, S. Stock

**Affiliations:** 1 Institute of Health Economics and Clinical Epidemiology, University Hospital of Cologne, Cologne, Germany; 2 Institute for Health and Nursing Science, Faculty of Medicine, Martin Luther University Halle-Wittenberg, Halle (Saale), Germany; 3 Department of Psychosomatics and Psychotherapy, Faculty of Medicine, University Hospital Cologne, University of Cologne, Cologne, Germany; 4 Center for Health Economics and Health Services Research, Schumpeter School of Business and Economics, University of Wuppertal, Wuppertal, Germany; 5 Center for Familial Breast and Ovarian Cancer, Center for Integrated Oncology (CIO), University of Cologne, Faculty of Medicine and University Hospital Cologne, Cologne, Germany

**Keywords:** Decision coaching, BRCA1 and BRCA2, Familial breast cancer, Familial ovarian cancer, Decision aid, Decision making, Patient-centered care, Shared decision-making

## Abstract

**Background:**

Female BRCA mutation carriers have an increased lifetime risk for breast and ovarian cancer compared to the general population. Women who carry this mutation have several options to deal with their cancer risk, such as risk-reducing surgeries or intensified breast cancer screening. Previous research has shown that preferences in this scenario are highly dependent on affected women’s personalities and value systems. To support these women in the decision-making process, a structured decision support consisting of decision coaching combined with a decision aid might be helpful.

**Methods/design:**

A randomized controlled trial will be conducted in order to compare usual care with structured decision support alongside usual care. The decision support program entails nurse-led decision coaching as well as an evidence-based patient decision aid. Nurses are qualified by a 4-day training program in informed decision-making and decision coaching. Six centers for Familial Breast and Ovarian Cancer in Germany will be included in the study, with a planned sample size of 398 women.

The primary outcome is the congruence between the preferred and the actual played role in the decision-making process as measured by the Control Preferences Scale. It is hypothesized that the structured decision support will enable women to play the preferred role in the decision-making process. Secondary outcomes include the knowledge and attitudes about preventive options, decisional conflict, depression and anxiety, coping self-efficacy, impact of event, and self-concept. A process evaluation will accompany the study.

**Discussion:**

The EDCP-BRCA study is the first study to implement and evaluate decision coaching combined with a decision aid for healthy BRCA mutation carriers worldwide.

**Trial registration {2a}:**

DRKS-ID: DRKS00015527. Registered 30 October 2019.

## Administrative information

Note: the numbers in curly brackets in this protocol refer to SPIRIT checklist item numbers. The order of the items has been modified to group similar items (see http://www.equator-network.org/reporting-guidelines/spirit-2013-statement-defining-standard-protocol-items-for-clinical-trials/).

**Table:** 

Title {1}	Implementation and evaluation of a nurse-led decision coaching program for healthy breast cancer susceptibility gene (BRCA1/2) mutation carriers: a study protocol for the randomized controlled EDCP-BRCA study
Trial registration {2a and 2b}.	DRKS-ID: DRKS00015527 (registered 30/10/2019)
Protocol version {3}	Version 1
Funding {4}	The study is fully funded by the Innovation Fond of German Federal Joint Committee. The study protocol has passed a peer-review selection process. (01VSF17043)
Author details {5a}	Isselhard A^1^, Töpper M^1^, Berger-Höger B^2^, Steckelberg A^2^, Fischer H^3^, Vitinius F^3^, Beifus K^4^, Köberlein-Neu J^4^, Wiedemann R^5^, Rhiem K^5^, Schmutzler R^5^, Stock S^1^ Author Affiliations 1 Institute of Health Economics and Clinical Epidemiology, University Hospital of Cologne 2 Institute for Health and Nursing Science, Faculty of Medicine, Martin Luther University Halle-Wittenberg, Halle (Saale) 3 Department of Psychosomatics and Psychotherapy, Faculty of Medicine, University Hospital Cologne, University of Cologne, Cologne, Germany 4 Center for Health Economics and Health Services Research, Schumpeter School of Business and Economics, University of Wuppertal, Wuppertal, Germany. 5 Center for Familial Breast and Ovarian Cancer, Center for Integrated Oncology (CIO), University of Cologne, Faculty of Medicine and University Hospital Cologne, Cologne, Germany
Name and contact information for the trial sponsor {5b}	Innovation Fond of German Federal Joint Committee Wegelystr. 8 10,623 Berlin
Role of sponsor {5c}	The design of the trial, the collection, analysis, and interpretation of data and the writing of this manuscript were not influenced by the funding body.

## Background

### Background and rationale {6a}

In Germany, around 70,000 women are diagnosed with breast cancer every year. An additional 7,200 women are diagnosed with ovarian cancer [1]. Approximately 30% of these new diagnoses have a family history of these cancers, a quarter of which are due to a pathological gene mutation in either the breast cancer type 1 or type 2 susceptibility genes (*BRCA1* and *BRCA2*, respectively*)*. *BRCA1* and *BRCA2* genes are tumor suppressor genes that produce proteins which are responsible for repairing DNA [2]. A mutation in either of these two genes has been found to significantly increase the risk of breast and ovarian cancer development, with an approximately 72% (95% confidence interval (CI) 65–79) or 69% (95% CI 61–77) cumulative lifetime risk (up to the age of 80 years) for breast cancer for *BRCA1* or *BRCA2* mutation carriers, respectively. *BRCA1* mutation carriers seem to be affected slightly more often and at a younger age than *BRCA2* mutation carriers. The cumulative lifetime risk for ovarian cancer is 44% (95% CI 36–53) for *BRCA1* and 17% (95% CI 11–25) for *BRCA2* mutation carriers [3]. In comparison, the lifetime risk for breast cancer in the general population is roughly 13%, while the risk for ovarian cancer is around 1%.

Several familial indications suggest genetic testing for healthy women, such as having two or more women with either breast or ovarian cancer, or having one woman with both cancers, or having only one woman with breast cancer who was younger than 36 years at diagnosis [4].

These differences in cancer risk and age at onset display the complexity of the genetic counseling provided for healthy *BRCA1/2* mutation carriers. In order to deal with the increased risk of developing cancer, healthy mutation carriers can choose between different options to reduce cancer incidence and mortality. The options are an intensified breast cancer-screening regimen, which includes biannual to annual magnetic resonance imaging (MRI) and mammography as well as semi-annual ultrasound examination, or prophylactic surgeries, such as bilateral mastectomy and/or salpingo-oophorectomy. While the intensified breast cancer screening does not reduce the likelihood of developing cancer, it detects breast cancers in stage 0 (carcinoma in situ) or stage 1 in approximately 80% of cases [5, 6]. However, there is a remaining risk of detecting cancer too late for effective treatment. In addition, particularly women with a *BRCA1* mutation develop triple-negative breast cancers in 75% of cases [7], meaning there is no expression of estrogen, progesterone, or human epidermal growth factor receptors [8]. These breast cancers require chemotherapy and are usually associated with poor prognosis [7, 8].

By contrast, women who have undergone risk-reducing bilateral mastectomies have been found to significantly reduce their breast cancer risk to a remaining risk of 5% on average [9]. One study reported that women with intact ovaries reduce their breast cancer risk through bilateral mastectomy by 90%, while women who had prior salpingo-oophorectomies reduced their risk by 95% [10], indicating that salpingo-oophorectomies have an impact on breast cancer risk. In fact, one study confirmed that salpingo-oophorectomies do indeed reduce breast cancer risk when performed premenopausally [11]. Additionally, salpingo-oophorectomies have been associated with an 80–90% reduction of ovarian cancer [12]. Some women decide to not take any action to deal with their individual cancer risk, in which case the lifetime risk remains unaffected.

For *BRCA1/2* mutation carriers, the decision between prophylactic surgeries and/or joining the screening program is based upon age of the woman, memories of family cancers, fertility and desire to have children, caring for children, close relationships, body image, ongoing risk and survival, among others [13–17]. For example, in one study, over a third of women who had undergone prophylactic mastectomy reported a decrease in their level of body satisfaction [17]. The same women showed significant decreases in their emotional concern about developing breast cancer. In case of the screening program, about 10% of MRI examinations result in unclear findings and require further imaging and examination [6]. This often results in high levels of anxiety in women [18] even though the majority (75%) of the results turn out non-cancerous [6, 19].

The examples above show the significant difficulty which healthy women with a *BRCA1/2* gene mutation face when encountering their breast cancer risk. Previous research with breast cancer patients has shown that the majority of patients prefer to be involved in medical-decision making [20, 21]. Not addressing these preferences can result in increased anxiety and decreased satisfaction [22]. In an international sample of breast cancer patients, Brown and colleagues [21] showed that while roughly 63% of patients prefer patient-centered or shared decision-making pre-consultation, only 54% experienced the decision-making as such, while 46% experienced the consultation as being “oncologist directed”. Furthermore the study showed that patients who were as involved as they anticipated, or received an even more patient-centered approach than they anticipated, reported higher satisfaction with the decision-making process, lower levels of decisional conflict, as well as greater satisfaction with the decision. This shows that patients should be actively involved in medical decision-making. The German Federal Ministry of Health picked up these findings and included them in the National Cancer Plan guideline, which now lists providing evidence-based patient information as well as patient involvement in medical decision-making as two of its goals [23]. Additionally, the German patient’s rights act and the German medical treatment guideline for breast cancer confirmed the patient’s rights of participation and informed decision-making. Informed decision-making implies that women are enabled to make their choices based on adequate knowledge about existing options and in congruence with their individual preferences [24, 25].

Two ways in which these goals can be accomplished in situations where more than one treatment option is available are evidence-based patient decision aids or decision coaching programs. Decision aids are evidence-based patient information that may be delivered in a variety of forms, such as web-based, paper-based, or video-based [26]. They have been shown to increase patients’ knowledge about treatment options, improve risk perception and support value-based, active decision-making [26]. Usually, they are used to supplement the physicians’ consultation since patients are often too overwhelmed by the diagnoses to fully absorb all information presented [27].

Decision coaching on the other hand refers to a consultation with a trained health-care professional in which all treatment options are discussed in a non-directive manner [28]. This exchange helps the patient to thoroughly understand the risks and benefits associated with each treatment decision, evaluate decisional needs, assess important personal values for the decision, as well as acquire strategies to communicate a decision to the physician to facilitate shared decision-making [28]. Decision coaching may vary by form (face-to-face, telephone call, or video call) or by person providing it (psychologist, nurse, pharmacist, social worker, or counselor among others). The person providing the decision coaching (decision coach) should be qualified in decision support and have sufficient expertise in the field of the decision. Additionally, contextual knowledge of the clinical surroundings simplifies the coordination with the physician after the decision coaching. Green and colleagues [29] showed that decision coaching could significantly improve knowledge about treatment options compared to usual care.

Both forms of decision support (decision aid and decision coaching) are often combined, for example, when the decision coach offers a decision aid as the basis of the decision coaching [30, 31]. In a systematic review significant differences were found regarding the outcomes knowledge about treatment options, perceived participation in decision-making, satisfaction with the decision-making process between the intervention group (IG; decision coaching and decision aid) and the usual care control group (CG) [26]. Moreover, in a trial that compared decision coaching combined with a decision aid with decision aid alone, patients who also received the decision coaching experienced a more active role in the decision-making process [32].

Internationally, only a few studies have explored decision support for *BRCA1/2*-positive women without breast cancer. One recent study pilot tested a paper-based decision aid and found it was well accepted by affected women as well was experts [33]. A randomized controlled trial (RCT) testing the effectiveness of a decision aid recruited women up to one month after receiving a positive *BRCA1/2* test result and were followed for 12 months [34]. It was found that women receiving the decision aid had significantly lower cancer-related distress at 6 and 12 months compared to the CG. Decision coaching has not yet been applied in *BRCA1/2*-positive women. However, a recent German study has shown that the extent of patient participation in treatment decision making was significantly higher in women with ductal carcinoma in situ who received nurse-led decision coaching combined with an evidence-based decision aid compared to standard care. In addition, only women in the IG made informed choices [35]. Despite these promising findings, decision support programs have not been systematically implemented in Germany. This study protocol describes a RCT evaluating structured decision support consisting of a decision aid and decision coaching for *BRCA1/2*-positive women with no prior history of breast or ovarian cancer.

### Objectives {7}

The EDCP-BRCA study aims to assess the effectiveness of decision coaching combined with a decision aid. The main hypothesis is that *BRCA1/2* mutation carriers who receive nurse-led decision coaching and a decision aid will show a higher congruence between the role they preferred to play in the decision-making process and the role they actually played. Secondly, we hypothesize that women who receive nurse-led decision coaching combined with a decision aid show higher satisfaction with the role they played in the decision-making process when compared to the control group. Additional objectives of the study are to evaluate whether decisional conflict and psychological burdens can be decreased and knowledge about the different preventive options can be increased.

Additionally, process evaluation will be conducted alongside the study to identify the barriers and facilitating factors of implementation. Secondly, the nurses and participating women are asked to share their experiences of the intervention. Finally, the process evaluation aims at investigating the underlying mechanisms of the intervention in relation to the context and interpreting summative results considering the impact of the intervention.

### Trial design {8}

The trial is designed as a multicenter superiority parallel group trial with a 1:1 allocation ratio. Randomization will take place on an individual basis.

## Methods/design

This study protocol is reported in accordance with the SPIRIT (Standard Protocol Items—Recommendations for Intervention Trials) criteria [36].

### Study setting {9}

The study will be conducted in six centers for Familial Breast and Ovarian Cancer in Germany (Cologne, Heidelberg, Kiel, Würzburg, Munich, and Dresden) which belong to the German Consortium for Familial Breast and Ovarian Cancer and are part of university hospitals. These clinics were chosen because they are the largest centers in terms of numbers of cases and performed genetic testing and they are spread throughout Germany.

### Eligibility criteria {10}

Women aged 25–60 years with diagnosed, clearly pathogenic *BRCA1/2* mutations and sufficient knowledge of the German language, who have not been diagnosed with breast or ovarian cancer, and who sign the consent form will meet the inclusion criteria.

Women with an unclear sequence variant in the *BRCA1/2* genes (variant of unclear significance (VUS) I-ARC class 3), women who have already been diagnosed with breast or ovarian cancer, women age under 25 years and over 60 years, with cognitive impairments and/or insufficient knowledge of the German language will be excluded from the study.

Nurses are eligible to lead the decision coaching if they have a specialized qualification as a breast care nurse or have other significant work experience in the field of gynecologic oncology. Additionally, they will complete training specifically developed to qualify nurses to lead decision coaching.

Physicians who will recruit women into the study are eligible to do so after having completed communication training (see the “Optimized standard care” section).

### Informed consent {26a}

Written informed consent will be obtained from study participants by the treating physician upon genetic counseling. Additionally, those nurses and physicians who will be interviewed for the formative evaluation will give informed consent prior to being interviewed.

### Additional consent provisions for collection and use of participant data and biological specimens in ancillary studies, if applicable {26b}

On the consent form, participants will be asked if they agree to use of their data should they choose to withdraw from the trial. Participants will also be asked for permission for the research team to share relevant data with people from the universities taking part in the research or from regulatory authorities, where relevant. This trial does not involve collecting biological specimens for storage.

### Intervention {11a}

#### Intervention group (IG)

Women in the intervention group will receive complex decision support intervention. This comprises (1) an evidence-based decision aid and (2) one or two structured nurse-led decision coaching sessions embedded in (3) optimized standard care.

##### Evidence-based decision aid

The evidence-based decision aid (DA) used in this study has been developed in a prior project funded by the NRW Centre for health Landeszentrum Gesundheit Nordrhein-Westfalen (LZG.NRW). Results of an RCT testing the effectiveness of the decision aid alone are currently pending. The aim of the DA is to enhance knowledge of the benefits and risks of the individual options (intensified breast screening, prophylactic surgery, and no action). The DA is based on evidence-based information and integrates the current findings of risk communication by using fact boxes and presenting risks as natural frequencies. It was developed by experts in the field and pilot tested by affected women before entering the RCT.

##### Decision coaching

Women in the IG will receive one or two (if required) 1-hour decision coaching sessions with a trained nurse supported by the DA. The nurse will, upon randomization in the IG, call these women to arrange an appointment for the decision coaching 2 weeks after being included in the study. The decision aid will be sent to the women by mail before the appointment.

The decision coaching is based on the Ottawa Decision Support Framework [37] and the nurse supports the decision-making process following these steps:

1. Clarification of the decision situation: What are the options? Where does the woman stand in her decision making process?
2. Identifying support needs: What does the woman know about her personal risks due to the *BRCA1/2* mutation and the prevention strategies?
3. Providing information and advice on the individual risks and prevention strategies according to the information of the evidence-based decision aid.
4. Support in clarifying individual values and preferences.
5. Support in weighing the different options.
6. Clarification of the practicability of the alternatives for action in the woman’s life context.
7. Communication of the decision.

The decision coaching is structured by patient decision guidance adapted to the special decisional needs of women with *BRCA1/2* mutation. Women and nurses can document the decision-making process within the decision guidance. In addition, the nurse will be prepared with fact sheets that provide essential information about the prevention strategies.

##### Optimized standard care

The centers of the German Consortium for Familial Breast and Ovarian Cancer already have, to varying degrees, an advisory infrastructure with training in risk communication and general communication techniques for the physicians in charge of counseling. In order to ensure the comparability of all centers, all physicians who supervise women participating in the study must have participated in 2.5-day communication skills training with a refresher session using actor patients, own difficult cases, role plays, and video tapes based on KoMPASS training [38]. The participating physicians will be trained in communication techniques such as the basics of counseling techniques, breaking bad news, dealing with difficult emotions, dealing with death and dying, using teach-back, and learning to communicate complex medical content in a comprehensible way. Physicians own challenging sample cases are used as a central basis for effective, learner-centered training (“experiential training”). The evaluation of these simulations takes place through the analysis of video recordings of the interactions.

### Explanation for choice of comparators {6b}

The comparator group in this trial (control group) will receive optimized standard care only (see the “Intervention group” section). Women will not be given any additional patient information and will not be invited for a coaching session. Standard care comprises at least one physician encounter in which women are informed about their diagnoses and the prevention options. In this study, women in the intervention as well as the CG will have at least one more physician consultation in which any open questions can be clarified and the woman is able to communicate her decision. This consultation can take place on site in the clinic or by phone.

### Strategies for implementation

#### Nurse training

Prior to providing the decision coaching, nurses will be trained in decision support with an adapted curriculum that has already been evaluated in German breast care centers [35, 39]. The training consists of two modules (module 1, 2.5 days; module 2, 1.5 days). Module 1 gives an overview of informed shared decision-making and the concept of evidence-based decision-making. Nurses acquire competence in risk communication and evidence-based patient information. In addition, nurses gain insight into the materials used within the decision coaching consultations (fact sheets, prompt cards, and decision guidance). Module 2 focuses on decision coaching skills, including training with actresses simulating real consultations. Additionally, the nurses are trained regarding basics of counseling techniques, dealing with difficult emotions, and using teach-back.

### Criteria for discontinuing or modifying allocated interventions {11b}

We do not expect the decision coaching to cause any adverse effects. Women are free to terminate their participation in the study at any point in time.

### Strategies to improve adherence to interventions {11c}

Women participating in the study who are randomized into the IG will be called to make an appointment for the decision coaching. Additionally, they will receive an invitation letter with the date and time of the decision coaching. If an appointment is missed, study participants will be called again and be asked to reschedule.

The study centers will be asked to report numbers of potential recruitments per week. This number will be compared to actual recruitments per week to ensure that all women are offered participation.

### Relevant concomitant care and interventions that are permitted or prohibited during the trial {11d}

The implementation of the intervention does not require alterations to the usual care pathways in both trial arms, including the use of any medication. All usual care pathways will continually be permitted.

### Outcomes {12}

The primary outcome of the study is the congruence between the preferred and actual role in the decision-making process as measured by the Control Preferences Scale (CPS) [40, 41]. Secondary outcomes are satisfaction with the actual role, decisional conflict (Decisional Conflict Scale (DCS)) [42, 43], knowledge and attitude towards preventions strategies (Multidimensional Measure of Informed Choice (MMIC)) [44, 45], stage of decision-making (Stage of Decision Making Scale (SDMS)) [46, 47], symptoms of anxiety and depression (Hospital Anxiety and Depression Scale (HADS)) [48, 49], coping self-efficacy (Coping Self-Efficacy Scale (CSES)) [50], subjective impact of the test result (Impact of Event Scale (IES)) [51, 52], and self-concept of *BRCA1/2* carriers (BRCA Self-Concept scale (BRCA-SC)) [53]. The instruments that will be used to measure these outcomes will be obtained at three different time points. Baseline measures will be obtained before randomization within a week of inclusion in the study, t1 measures will be obtained 12 weeks after inclusion, and t2 at 6 months after inclusion in the study. Table 1 displays the outcome parameters and time points for outcome measurements.

**Table 1:** Outcome parameters

	Study period						
	Enrolment		Allocation	Post-allocation			
Timepoint		Directly after inclusion		1 week after inclusion	Within 3 weeks after inclusion	12 weeks after inclusion	6 months after inclusion
Enrolment							
Eligibility screen	X						
Informed consent	X						
Allocation			X				
Interventions							
Decision aid				X			
Decision coaching					X		
Assessments		t0				t1	t2
Control Preferences Scale							
Desired role		X					
Actual role						X	
Satisfaction						X	
Decisional Conflict Scale		X				X	X
Multidimensional Measure of Informed Choice							
Attitudes		X				X	X
Knowledge		X				X	X
Stage of Decision Making Scale		X				X	X
Hospital Anxiety and Depression Scale		X				X	X
Coping Self-Efficacy Scale		X				X	X
Impact of Event Scale		X				X	X
BRCA Self-Concept Scale		X				X	X

#### Congruency between desired and actual role in the decision process and satisfaction with the actual role

The congruence between the desired and actual role in the decision-making process and satisfaction with the actual role played is assessed by the Control Preferences Scale (CPS) [40, 41]. The CPS was designed to measure the degree of control that patients want to assume in physician–patient interactions and has often been used in studies on decision support. The items were adapted to the decision situation of *BRCA1/2* mutation carriers by replacing the term “treatment” with “prevention strategy”. The desired role will be measured at baseline and the actual role at T2 (12 weeks after inclusion). The difference between these two measurements reflects the congruence between desired and actual role. One item will be added to the CPS to measure satisfaction with the actual role played at T2.

#### Decisional conflict

Decisional conflict will be assessed using the Decisional Conflict Scale (DCS) [42, 43]. It captures uncertainty in health-related decisions. The DCS comprises 16 items and includes five subscales: information, personal uncertainty, clarification of values, support or pressure from others, and perception of the quality of the decision process. The items are scored on a five-point Likert scale (1 = strongly agree to 5 = strongly disagree). The German translation of the DCS showed good psychometric properties [43]. The DCS will be used at T1, T2, and T3.

#### Attitude and knowledge

Attitudes towards prevention strategies will be measured by the attitudes subscale of the Multidimensional Measure of Informed Choice (MMIC) [44, 45]. The MMIC requires generating items to measure knowledge in the field of interest, while the attitude items are generic and usable for all types of scenarios. Knowledge about the different prevention strategies available to *BRCA1/2* mutation carriers will be tested using a set of 15 items developed by experts in the field developed in a prior study. For each item, the participant has to indicate whether a statement is correct or false. The knowledge test includes questions about lifetime cancer risk for mutation carriers, benefits and harms about each preventive option, as well as lifestyle-related questions. A choice is rated as informed as soon as women have adequate knowledge (at least eight correct knowledge items) about the option and a positive attitude toward the chosen option.

#### Stage of decision-making

To assess the stage of decision-making we used an adapted stage of decision-making (SDMS) [46]. The German Translation and adaption consists of one single item and four response options from “I haven’t begun to think about the choices” to “I have already made my choice” [47].

#### Anxiety and depression

Symptoms of anxiety and depression will be measured with the Hospital Anxiety and Depression Scale (HADS) [48, 49]. The HADS is a widely used instrument that comprises two scales, one of which assesses symptoms of anxiety, the other symptoms of depression. The 14 items are rated on a four-point Likert scale. The HADS shows good psychometric properties, with most studies reporting an internal consistency of Cronbachs alpha = 0.8 or higher.

#### Coping self-efficacy

Coping self-efficacy will be measured using the CSES, which is a 13-item measure of an individual’s confidence to cope with life challenges [50]. The women are asked to indicate how confident they are to show different coping behaviors in situations where they are confronted with problems. The items are scored on an 11-point Likert scale (0 = ‘cannot do at all’, 5 = ‘moderately certain can do’ and 10 = ‘certain can do’).

#### Impact of genetic test result

To assess the subjective impact of the test result we will use the IES (revised version) [51, 52]. The scale covers three dimensions of symptoms often reported after trauma: intrusion, avoidance, and persistent hyperarousal. The women will be asked to rate the experience of 22 symptoms of traumatic stress over the past week. Response options are ‘not at all’, ‘rarely’, ‘sometimes’, and ‘often’.

#### Self-concept

The BRCA-SC will be used to assess the changes experienced by *BRCA1/2* carriers in their perceptions of themselves [53]. The BRCA-SC is a 17-item scale with response options ranging from 1 = ‘strongly disagree’ to 7 = ‘strongly agree’. It consists of three subscales for stigma, vulnerability, and mastery. The questionnaire was translated into German by the research team following the TRAPD methodology [54].

### Participant timeline {13}

Women who are potentially eligible for the study but have not yet been tested for a *BRCA1/2* gene mutation will be informed about the study at their appointment for collecting the blood sample for genetic testing. Approximately 4 weeks after collection of the blood sample, the women have an appointment to discuss the test results with their physicians. During this appointment, only women with a positive *BRCA1/2* test result will be invited to participate in the study, and their informed consent will be obtained. Women who have previously been tested positive for *BRCA1/2* at a different facility from the six study centers will be invited to participate during their first consultation at the respective study center. Women who are *BRCA1/2* mutation carriers and have already joined the intensified breast-cancer screening program will be invited to participate during their semi-annual appointment. Physicians will complete a recruitment form in which they can also document reasons for non-participation.

#### Baseline data and allocation

After recruitment, women will be given the first questionnaire to take home. After sending back their baseline questionnaire, they will be randomized into one of the two groups. Randomization will be performed on a patient basis after the consultation or breast cancer screening, in order to ensure equal treatments by the physician in both groups.

#### Intervention group

Women in the IG will be notified shortly after returning their baseline questionnaire. They will be invited to schedule an appointment with the qualified nurse for the decision coaching. Additionally, they will be sent the patient decision aid. The decision coaching will be scheduled to take place within 3 weeks of giving informed consent.

#### Second physician encounter

Both groups will receive a second physician encounter. In the IG, women will communicate their preferred choice to the physician. In the CG, women will have an additional opportunity to ask questions. This was designed to ensure equality of treatment in both groups. The second physician encounter may take place over the phone.

#### First and second follow-up

**Fig. 1:**
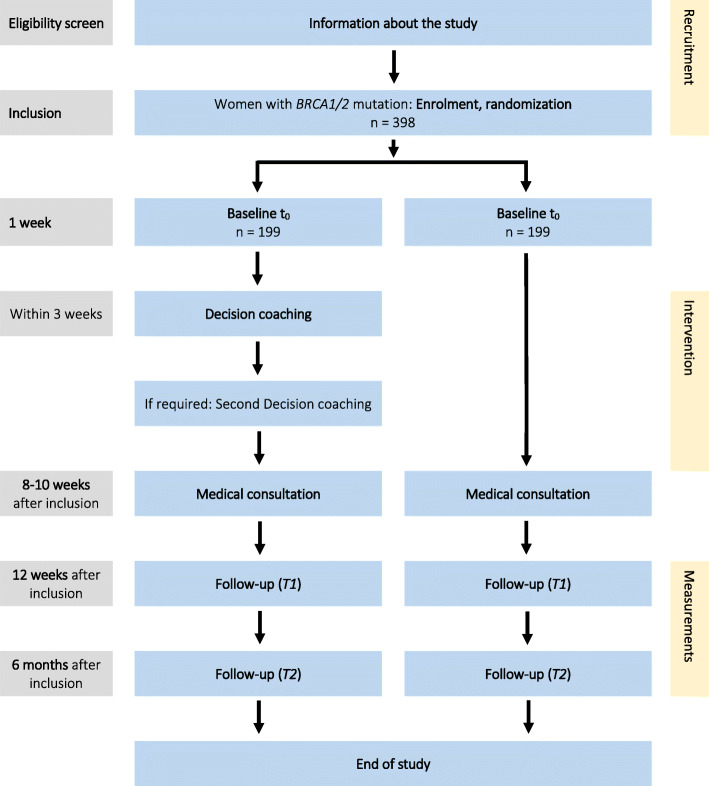
Participant timeline

Women in both groups will receive the first and second follow-up questionnaire after 12 weeks and 6 months of genetic diagnosis, respectively. Women in the CG will be informed about the group allocation during the scheduling of the second physician encounter, so that they no longer wait for an appointment for the decision coaching. The questionnaires will be sent by mail with a prepaid envelope attached to return the questionnaire. For a schematic overview of the participant timeline see Fig. 1.

### Sample size {14}

The sample size calculation for the summative evaluation is based on an assumed small to moderate effect of the intervention (Cohen’s *d* = 0.3) in a two-sided *t*-test between two groups [28]. It accounts for a drop-out rate of 30%, a power of 0.8 and an alpha error of 0.05. Based on these numbers, the software G*Power [55, 56] yielded a total *n* = 398 (199 participants per group).

### Recruitment {15}

All women who have appointments at one of the six study centers during the recruitment period (November 2019–January 2021) and are potentially eligible for the study will be invited to participate. Study centers have a sufficient number of genetic tests every month to guarantee sufficient enrollment in the study.

## Assignment of interventions

### Sequence generation {16a}

The study IDs are assigned to the IG or CG by means of a randomized allocation sequence that was generated using computer-generated random numbers.

### Allocation concealment mechanism {16b}

Only the members of the research team at the data evaluation institute know the allocation of the study IDs to the groups. The list with study IDs and group allocations is password protected and only accessible by the research team.

### Implementation {16c}

At baseline, each woman is assigned a study ID by means of prenumbered envelopes. Only after the women have sent back the baseline questionnaire to the research team will the respective clinic in which the women were recruited will be informed which group the study ID was assigned to via an encrypted e-mail by the research team. The six study centers have no means of accessing the group allocations before baseline measures are obtained.

### Blinding {17a}

Due to the nature of the study, study participants and the treating physicians cannot be blinded. The members of the research team responsible for analyzing the data will be blinded to group allocation by removing study IDs of the data set prior to data analysis.

### Unblinding {17b}

The design is open label with only data analysts being blinded so unblinding will not occur.

## Data collection, management, and analysis

### Data collection methods {18a}

The questionnaire data will be entered by the research team at the University Hospital of Cologne who are not involved in consultation and recruitment of patients.

### Participant retention and complete follow-up {18b}

Study participants in both intervention and control groups will receive postal reminders when questionnaires have not been sent back in 1 week and over the phone reminders when questionnaires have not been sent back in 2 weeks to promote participant retention and minimize dropouts.

### Data management {19}

The software Remark office will be used to extract pseudonymized data from the questionnaire with two independent members of the research team manually verifying the extracted data. The paper-based questionnaires will be kept in locked storage in accordance with German law. The extracted data will be stored in password-protected files on password-protected computers only accessible by the research team.

### Confidentiality {27}

Personal information and the data obtained by the participants will be kept and stored separately. Only the recruiting physicians will have a list that allows matching of study ID and personal information. This list will be kept strictly confidential and only be accessible by recruiting physicians in their respective study center. The research team will only receive pseudonymized questionnaire data.

### Biological specimens {33}

No biological specimens will be collected.

## Statistical methods

### Statistical methods {20a}

Baseline data will be analyzed to guarantee comparability of both groups. Mean differences of the primary outcome in both groups (congruence between preferred role at baseline and actual role in the decision-making process at t1) will be compared using an independent samples one-sided *t*-test with the group allocation as the independent variable. All secondary outcomes will be analyzed via independent samples *t*-tests, Chi-square tests, or nonparametric tests in case of non-normality. Beyond group allocation, demographic data will be used as the independent variable. All data analysis will be performed with SPSS and R.

### Interim analyses and stopping guidelines {21b}

Interim analyses are not planned. No problems detrimental to the participants are anticipated; therefore, this trial has no formal stopping guidelines.

### Methods for any additional analyses {20b}

#### Formative evaluation

A comprehensive process evaluation from the beginning to the end of the study is necessary to understand the underlying mechanisms of the intervention, the contextual factors influencing outcome development, to ensure the generalizability of the study results, and to improve future implementations.

Although this study is no cluster-randomized trial, the formative evaluation follows Grant’s framework for designing process evaluations [57]. The framework was adapted to our study. Especially the following elements will be considered: recruitment (difference between participating and non-participating clinics; recruitment of women), implementation of the intervention (delivery to clinics and women: fidelity, dose, adaptations made, reach), and responses of the healthcare professionals (physicians and nurses) as well as included women. Furthermore, we will integrate the contextual perspective. Therefore, we will explore the context in which the trial is being conducted and examine how the intervention is introduced. This adoption of a systems lens may enable us to look at concepts like feasibility or acceptability in dynamic terms and investigate how clinic responses to interventions change over time, rather than viewing context as background noise [58]. The outcome will be interpreted in the light of these identified processes.

A mixed-methods approach will be used to collect data alongside the RCT. Quantitative data will be analyzed using descriptive statistics, qualitative data from semi-structured interviews will be tape-recorded and transcribed verbatim. Further, we will generate qualitative field notes that capture observations made during study events (e.g., training sessions, project meetings). Transcribed data, data form field notes, as well as data from open-ended questions in the questionnaires and monthly reports of the trained nurses will be imported into MAXQDA software (VERBI GmbH, Berlin, Germany) and analyzed using principles of content analysis [59].

A summary of the central questions in the process evaluation and data collection methods are presented in Additional file 1.

### Protocol non-adherence and missing data {20c}

Data analysis will be conducted according to the intention-to-treat method. Imputation of missing data is not planned. Additionally, a per-protocol analysis is planned as a sensitivity analysis to exclude participants who did not comply with the allocated intervention by missing the decision coaching or by failing to return all questionnaires.

### Public access {31c}

The datasets analyzed during the current study are available from the corresponding author on reasonable request.

## Oversight and monitoring

### Roles and responsibilities {5d}

The steering committee of the trial consists of all authors of this paper. The steering committee will meet biweekly and monitor the trial progress. Quarterly progress reports are given to the funding agency. KR, RW, and RS will oversee participant enrollment. AI, MT, and SST will monitor participant retention, allocation of participants to groups, and adherence to trial interventions.

### Data monitoring {21a}

The data monitoring committee consists of members of the research team and a statistician, who monitor the data independently from the funding agency.

### Adverse events {22}

Evidence on decision coaching trials suggests that adverse events or serious adverse events are not anticipated [28]. Minor adverse events are likewise not anticipated but will be reported to the relevant regulatory bodies as required.

### Auditing trial conduct {23}

The trial steering committee will continually review trial conduct. Formal audits are not planned.

### Protocol amendments {25}

Changes in the study protocol will first be communicated to the funding agency. A revised study protocol will then be sent to the study centers. The clinical trial registry entry will be updated upon changes in the study protocol.

## Dissemination plans {31a}

Results of this trial will be published in scientific journals.

## Discussion

The EDCP-BRCA study is the first study to systematically implement and evaluate structured decision support in healthy *BRCA1/2* mutation carriers worldwide. The purpose of this study is to demonstrate that decision coaching combined with an evidence-based decision aid can improve patient-reported outcomes such as congruence of preferred and actual role in decision-making and satisfaction with the role in the decision-making process. Additional secondary outcomes include improvement in decisional conflict, increased knowledge about prevention options, and lower psychological distress. Another important target of this intervention is to investigate whether structured decision support can be integrated well into current usual care.

Three limitations of the study need to be addressed. First of all, women are informed about the nature of the study and the possibility of receiving decision support. If women are then randomized into the CG and are informed that they do not receive such additional support, there is an increased risk of dropping out of the study. Secondly, since *BRCA1/2* mutations run in families, there is no possibility to prevent family members from entering the study. This could result in some sort of contamination if two siblings are randomized into two different groups. This contamination effect will be analyzed with a family code that is assigned to each family in the German centers for breast and ovarian cancer. Additionally, women in the IG and CG could get in contact online or via self-help groups. Furthermore, due to counseling patients in the IG and the CG, a training effect of physicians might occur and therefore a contamination between IG and CG. Lastly, the intervention is of a complex nature, which includes a decision aid and decision coaching embedded in an optimized standard care setting. Such interventions have the inherent difficulty of attributing an overall effect to one of the components. For this reason, a process evaluation alongside the trial aims to untangle all components of the intervention as well as identify facilitating and inhibitory factors in the implementation.

## Trial status

Study protocol version 1 (06/01/2020)

The recruitment of study participants has started in November 2019 and will continue until January 2021.

## Supplementary information

**Additional file 1.** Characteristics of the planned intervention and implementation strategy. (modified according to Enola K. Proctor, Byron J. Powell, and J. Curtis McMillen. “Implementation strategies: recommendations for specifying and reporting.” Implementation Science 8.1 (2013): 139.)

**Additional file 2.** Summary of the process evaluation.

## Data Availability

The data set will be available to all principal investigators. The datasets generated for the current study will not be publicly available but will be available from the corresponding author on reasonable request.

## Abbreviations

*BRCA1/2*: Breast cancer susceptibility gene 1/2
CI: Confidence interval
IG: Intervention group
CG: Control group
