# Supplementary material for: Implementation and evaluation of a nurse-led decision-coaching program for healthy breast cancer susceptibility gene (BRCA1/2) mutation carriers: a study protocol for the randomized controlled EDCP-BRCA study
Source: Trials. 2020 Jun 8;21:501. doi: 10.1186/s13063-020-04431-x (PMC7278068; doi:10.1186/s13063-020-04431-x)
Supplement: Supplementary file 1 — Additional file 1. Characteristics of the planned intervention and implementation strategy. (modified according to Enola K. Proctor, Byron J. Powell, and J. Curtis McMillen. “Implementation strategies: recommendations for specifying and reporting.” Implementation Science 8.1 (2013): 139.) [file 13063_2020_4431_MOESM1_ESM.pdf]

Additional File 1: Characteristics of the planned intervention and implementation strategy
(modified according to Proctor, Enola K., Byron J. Powell, and J. Curtis McMillen. "Implementation strategies: recommendations for specifying and reporting." Implementation Science 8.1 (2013): 139.)

	Training for nurses	Decision coaching for women	Provision of a decision aid	Supervision of trained nurses	Supportive material for trained nurses	Supportive material for process organization
**Actor(s):** Who delivers?	Research team	Trained nurses	Trained nurses	Research team involved in training sessions	Research team, trained nurses	Research team, clinical staff
**Action:** What is delivered?	Decision coaching training for nurses	Decision coaching	Dissemination and use of a decision aid	Supervision and individual coaching for nurses	Supportive material for coaching sessions (e.g. decision path, fact sheets)	Supportive material enabling a structured organization of nurse-physician interaction and standardized documentation
**Target(s) of action:** Who receives actions?	(Breast care) nurses of the participating study centers	Women of the intervention group being treated in the participating clinics	Women of the intervention group being treated in the participating clinics	Trained nurses	Women of the intervention group being treated in the participating clinics	Clinical staff involved in treatment of the intervention group
**Temporality:** When is the action delivered?	At the beginning of the implementation phase	At least within 3 weeks after inclusion	After communication of the test results and randomization	After the first two decision coachings	During coaching sessions and their wrap-up	during implementation phase and intervention period
**Dose:** How much will be delivered?	According to training status 1 or 2 modules;<br><br>module 1: first day 4.5h, second day - 8h, third day – 8h<br>module 2 after 2-4 weeks: First day 4.5h, second day 8h	At least one decision coaching: first coaching: 40-60 min, second coaching if required: individual dose	Initial setup after randomization, postal delivery	On request	Demand-oriented use during coaching sessions and for an individual wrap-up at home	Presentation of supportive material during initiation of the study centers and training sessions for nurses
**Theory:** What is the underlying theory?	Theory of planned behavior	Theory of planned behavior, Ottawa Decision Support Framework	Ottawa Decision Support Framework	Theory of planned behavior	Theory of planned behavior	Theory of planned behavior
**Outcome:** Which outcome is addressed?	Uptake, Penetration/Reach, Fidelity, Acceptability	Service outcomes (Effectiveness, Patient-centeredness), Client Outcomes (Satisfaction)	Service outcomes (Effectiveness, Patient-centeredness), Client Outcomes (Satisfaction)	Fidelity, Sustainability	Service outcomes (Effectiveness, Patient-centeredness), Client Outcomes (Satisfaction)	Uptake, Appropriateness (perceived fit), Penetration/Reach, Sustainability
**i-PARIHS domain:** Which domain of the i-PARIHS framework is addressed?	„Recipients“ (Professional level)	„Recipients“ (Patient level)	„Recipients“ (Patient level)	„Recipients“ (Professional level)	„Innovation“ (Usability/feasibility), „Recipients“ (Patient level)	“Context” (local and organizational level), “Innovation” (Usability/feasibility), “Recipients” (Professional level- teamwork)

Note: grey columns summarize intervention elements, white columns give an overview on the implementation strategy
