# Supplementary material for: Implementation and evaluation of a nurse-led decision-coaching program for healthy breast cancer susceptibility gene (BRCA1/2) mutation carriers: a study protocol for the randomized controlled EDCP-BRCA study
Source: Trials. 2020 Jun 8;21:501. doi: 10.1186/s13063-020-04431-x (PMC7278068; doi:10.1186/s13063-020-04431-x)
Supplement: Supplementary file 2 — Additional file 2. Summary of the process evaluation. [file 13063_2020_4431_MOESM2_ESM.pdf]

Additional File 2: Summary of the process evaluation

Domain	Focus	Data collection format	Measurement point and informant	study group
Context	Study center:<br>• Clinic core: Resources, organizational structure and functional processes<br>• Adaptive reserve: Culture and Climate (in particular, innovation climate)<br><br>Physicians and nurses:<br>• experiences and awareness,<br>• general knowledge,<br>• qualification;<br>• self-efficacy and general attitudes<br><br>Participating women:<br>• sociodemographic characteristics,<br>• family history,<br>• general attitudes,<br>• health literacy,<br>• trust,<br>• self-efficacy and optimism<br><br>Intervention characteristics:<br>• complexity,<br>• compatibility<br>• relative advantage	semi-structured interviews,<br><br>standardized questionnaire	Study center (key informant), physicians and nurses: during initiation period of the study center<br><br>Participating women: Baseline (T0)	 <br><br><br>IG/CG
Recruitment of clinics	• organizational structure and functional processes of non-participating clinics	standardized documentation form	during initiation period of the study center	not applicable
Recruitment of women	• recruitment procedure,<br>• reasons for participation/non-participation	standardized documentation form	during recruitment process	IG/CG

Delivery to clinics	• fidelity: Was the implementation strategy delivered to the clinics as planned?<br>• dose: How much of the implementation strategy was delivered?<br>• adaptations: How much was the implementation strategy adapted by the clinics?<br>• reach: Has everyone been reached by the implementation strategy, who is important for the delivery of the intervention?	extraction of training protocols,<br><br>standardized questionnaire,<br><br>semi-structured interviews	training staff, nurses: during training sessions and six months after the last training session	not applicable
Delivery to women	• fidelity: Was the intervention delivered to women as planned?<br>• dose: How much of the intervention was delivered?<br>• adaptations: How much was the intervention adapted by the clinics?<br>• reach: Has everyone reached?	video- and/or audiotapes of randomly selected coaching sessions,<br><br>standardized documentation form,<br><br>copy of the patient decision guidance,<br><br>standardized questionnaire,<br><br>semi-structured trainer interview	nurses: continuously during study,<br><br>women: first follow-up (T1)<br><br>trainer: at the end of the supervision period	IG
response of clusters	• attitude of the clinics (organizational view, nurses and physicians) regarding the intervention and implementation strategy,<br>• adoption/uptake of the intervention and implementation strategy (how?),<br>• integration into daily routine (how?),<br>• changes in clinical behavior and attitudes	standardized documentation form,<br><br>semi-structured interview	nurses: continuously during study and at the end<br><br>Study center (key informant) and physicians: at the end	not applicable
response of women	• attitude about the decision coaching,<br>• use of coaching sessions and decision aid,<br>• satisfaction,<br>• decision process (e.g. second opinion, confidence about decision)	standardized questionnaire,<br><br>semi-structured interview	women: first (T1) and second follow-up (T2)<br><br>women: after the second follow-up (T2)	IG
Maintenance	• experienced process normalization,<br>• level of institutionalization,<br>• reflection of barriers and facilitators	standardized questionnaire,<br><br>semi-structured interview	nurses and key informants: at the end of the study	IG

			women: after the second follow-up (T2)	
Unintended consequences	• reflection of barriers and facilitators,<br>• unintended consequences in terms of work load,<br>• unintended processual changes,<br>• unintended consequence on women level (e.g. fear, uncertainty)	semi-structured interview	nurses and key informants: at the end of the study,<br><br>women: after the second follow-up (T2)	IG
Theories and frameworks	• impact of the intervention on decision making,<br>• Integration in daily routine and process normalization,<br>• Characteristics of the intervention,<br>• Implementation process	Theory of planned behavior,<br>Process normalization theory,<br><br>Rogers “Diffusion of innovations”,<br>PARIHS-Framework		
